# The increased purchase of asthma medication for individuals born preterm seems to wane with age: A register-based longitudinal national cohort study

**DOI:** 10.1371/journal.pone.0199884

**Published:** 2018-07-05

**Authors:** Anne Louise de Barros Damgaard, Rasmus Gregersen, Theis Lange, Frederik Buchvald, Bo Mølholm Hansen, Gorm Greisen

**Affiliations:** 1 Department of Neonatology, Copenhagen University Hospital Rigshospitalet, Copenhagen, Denmark; 2 Center for Statistical Science, Peking University, Beijing, China; 3 Department of Biostatistics, University of Copenhagen, Copenhagen, Denmark; 4 Pulmonary Service, Department of Pediatrics, Copenhagen University Hospital Rigshospitalet, Copenhagen, Denmark; 5 Neonatology Service, Department of Pediatrics, Copenhagen University Hospital Herlev, Herlev, Denmark; Centre Hospitalier Universitaire Vaudois, FRANCE

## Abstract

**Introduction:**

Preterm birth is associated with increased risk of respiratory symptoms in childhood, often treated with asthma medication. We designed a follow-up study to previous research and investigated whether the association of gestational age with purchasing asthma medication diminishes in adulthood.

**Methods:**

We conducted a register-based study of a national cohort of all infants born in Denmark in 1980–2009 evaluating longitudinal data on individually prescribed asthma medication (both inhaled ß-2 receptor agonist and different controller treatment over 2-year periods) available from 1995–2011. We analyzed the effect of gestational age considering age, birth year, and perinatal variables using logistic regression with a Generalized Estimating Equations model. All data were unambiguously linked through the Civil Registration System.

**Results:**

We included 1,819,743 individuals in our study population. We found an inverse dose-response relationship between gestational age and asthma medication in earlier age-groups with a gradual decrease in odds ratios with increasing age and loss of statistical significance in early adulthood (18–31 years). For our oldest generations, there was a significant effect of gestational age (p-value = 0.04), which became insignificant when adjusting for confounding and mediating factors (p = 0.44). There were significant interactions between gestational age and age (p<0.0001) and gestational age and birth year, but these were most important during childhood (0–11 years) and for our youngest generations (born after 1995).

**Conclusion:**

The strong association between gestational age and purchase of prescription asthma medication weakens with age into early adulthood, in consistence with the results from our previous study. The risk for purchasing medication to treat asthma-like symptoms was higher in more recent birth years, but the effect of gestational age was small beyond 11 years of age. Gestational age *per se* did not seem to be significant for the development of asthma-like symptoms: most of its effect could be explained by other perinatal factors.

## Introduction

Individuals born preterm experience increased respiratory symptoms later in life [1], often reported and treated as asthma, although the underlying pathophysiologic pathways might prove to be quite different [2]. This is documented by an increased use of prescription asthma medication—especially during early childhood—compared to their peers born at term [3–5]. In a large cross-sectional register study, we previously demonstrated a clear inverse dose-response relationship with decreasing gestational age (GA) but the effect seemed to diminish with age and lose significance in adulthood [5].

Asthma prevalence in the general population, however, is steadily increasing [6], and the chances of survival for the most ill and most immature infants have increased [7]. Thus, the weaker association between GA and use of prescription asthma medication in the older cohorts of our previous study could partly be attributable to the ‘healthy survivor’ effect, i.e. the older generations of children born preterm may simply be at lower risk of developing asthmatic symptoms requiring treatment with asthma medication, since the more vulnerable newborns were less likely to survive. This confounding by time period is often referred to as a cohort effect or generations effect [8]. Our previous findings [5] were limited by the risk of such a cohort effect and the cross-sectional design of that study did not allow a distinction between an actual decrease of the effect of GA and individuals who were simply born with a lower risk of asthma in adulthood.

Most follow up studies on respiratory sequelae after prematurity are cross-sectional and longitudinal trends from early childhood until adulthood are missing [9,10]. However, longitudinal data, repeated measurements on the same individuals and corresponding analyses are required to reliably reveal the true trends in use of asthma medication throughout childhood, adolescence and young adulthood and to demonstrate whether the association actually weakens over time. Therefore, we conducted this longitudinal study following birth year cohorts over time, their individual and repeated use of prescription asthma medication and its association with gestational age, under the hypothesis that the association found in early childhood does not persist into adulthood.

## Material and methods

### Data sources, access and ethics

We conducted a retrospective longitudinal observational register-based study of a national cohort of all infants born in Denmark in 1980–2009, created from a database used in a previous study [5]. The database was formed by linking all social and health entries from the Medical Birth Registry, the National Patient Register, the Register of Education of the Population and the Cause of Death Register to individual Central Personal Registration (CPR) numbers, which were then anonymized before access was given to the researchers. The Danish registers are consistently and unambiguously linked on an individual basis as a result of the Civil Registration System, where a unique CPR number is assigned to each individual at birth and used for all register entries of social and health services. The data sources for the Danish registers, their uses and their limitations have previously been thoroughly described [5,11–13]. The database was fully accessible to the researchers in its anonymized form, and the study has been approved by the Danish Data Protection Agency and Danish Health and Medicines Authority (Jr.no 2007-41-0806). Danish law does not require individual ethical approval for this kind of study. The anonymized data are available from Statistics Denmark. Access can be granted to Danish research environments that are pre-approved by Statistics Denmark or to foreign researchers with an affiliation to a Danish authorized environment. Whilst we cannot guarantee such an access, we can assist interested institutions in any request in this context. We have followed the Reporting of studies Conducted using Observational Routinely-collected Data (RECORD) [14] guidelines and a RECORD statement can be found in S1 Table.

### Study population

The study population was based on a cohort including all registered births in Denmark during the period 01.01.1980–31.12.2009 from the Medical Birth Registry. We excluded individuals who were stillborn, who had missing data on GA, gender or birth weight, or who had abnormal birth weight values defined as differing more than +/- 5 standard deviations (SD) from reference mean birth weights [15]. In addition, birth weight was set as missing if inferior to 100 g or superior to 9000 g and GA was set as missing outside the 23–45 weeks’ range, both under assumption of misclassification.

### Outcome

Data from the Danish National Prescription Registry provided the basis of our outcome assessment. The registry contains data on all purchases of prescription medication from all non-hospital pharmacies available during the period 1995–2011 [16,17]. The outcome, prescription asthma medication, was defined from Anatomical Therapeutic Chemical (ATC) codes in agreement with our previous study [5] as either:

- a combination of at least one purchase of inhaled selective ß-2 receptor agonist (R03AC) and at least two purchases of one of the following other drugs for obstructive airway disease: inhaled glucocorticoids (R03BA), inhaled anticholinergics (R03BB), teophyllins (R03DA), oral leukotriene-receptor antagonists (R03DC), systemic steroid (H02AB) or
- at least two purchases of a combination inhaler (R03AK, R03AL).

The outcome was evaluated from every even birthday starting from 0 years and in the following 2-year period (0–1, 2–3 years etc.). Only complete 2-year periods within the time frame 1995–2011 were evaluated. The 2-year period was also censored if the follow-up was not complete due to either death before or during the period or due to residence abroad at the start of the period or emigration during the 2-year period.

### Covariates

- GA: The main exposure of interest was gestational age in full weeks and 4 main groups were considered: term with GA ≥ 37, moderately preterm with GA 32–36, very preterm with GA 28–31 and extremely preterm with GA 23–27 weeks. GA was modelled as an ordinal variable with 4 levels from term to extremely preterm. Thus, the resulting ORs are for a one unit increase in prematurity (e.g. from GA group 32–36 to group 28–31 weeks), with the term group as the reference. This approach was chosen based on superior goodness-of-fit statistics compared with a model treating GA as a categorical variable.
- Age groups: Each 2-year observation period was categorized according to the following age groups: 0–1 (infants), 2–5 (preschoolers), 6–11 (school-aged children), 12–17 (adolescents), 18–23 and 24–31 years (adults) under parametric assumptions.
- Birth year: grouped into 1980–1984, 1985–1989, 1990–1994, 1995–1999, 2000–2004 and 2005–2009 under parametric assumptions.

In addition, we included the following variables with known associations with preterm birth and asthma in the analysis:

- Birth weight (BW): Small-for-gestational-age (SGA) was defined as a BW < -2 SD from Marsál’s reference value [15].
- Sex.
- Firstborn.
- Multiple birth.
- Delivery method: vaginal birth or cesarean section (emergency and elective).
- Maternal educational level: evaluated at time of birth and divided into three groups per the International Standard Classification of Education 2011 (ISCED) [18]. Group 1 included ISCED levels 0–2, group 2 levels 3–4 and group 3 levels 5–8. When information on maternal educational level was missing, data on paternal educational level were used instead.
- Maternal asthma and atopy medication: Asthma medication followed the same definition as the outcome and was evaluated in a 2-year period prior to birth. Since data were only available from 1995, for births before 1997, a 2-year period beginning in 1995 was used to supply data as close to birth as possible. Atopy medication was defined as at least one purchase of prescribed (ATC-codes): nasal steroid spray (R01AD), facial steroid cream group I (D07AA), steroid cream group III (D07AC) during the same 2-year period.
- Neonatal respiratory morbidity: assessed from admission data during the first 6 months after birth. The diagnoses used for acute neonatal respiratory disease were (International Classification of Diseases codes): respiratory distress and transitory tachypnea of the neonate (ICD-8 776.290/ICD-10 P22.1 and P22.8), respiratory distress syndrome (ICD-8 776.190/ICD-10 P22.0) and neonatal emphysema (ICD-8 776.200/ICD-10 P25.0). The diagnoses included for chronic neonatal respiratory disease were pulmonary fibrosis (ICD-8 517.010) before 1994 and bronchopulmonary dysplasia (BPD) (ICD-10 P27.1) after the 1994 classification system change.
- Maternal smoking during pregnancy: self-reported at prenatal visits. The variable was available from 1991 (only evaluated in a sub-group analysis).

### Statistical analyses

We analyzed our primary outcome using a logistic regression on the binomial asthma medication outcome. All valid outcome measurements from all individuals were analyzed together, and intra-person dependence was accounted for by using Generalized Estimating Equations (GEE) with an autoregressive working correlation matrix. Thereby, the GEE model allows for several measurements on the same individual to be analyzed together—which is not normally possible due to assumptions of independency—while correcting for potential correlation between measurements on the same individual. The repeated measures allow for an individual follow-up on a potentially decreasing asthma medication purchase over time. Analyses were carried out in two different models:

- Model 1: adjusted for GA, age, year of birth and both interactions between GA and age groups and GA and birth year groups respectively.
- Model 2: as model 1 + variables present at birth (SGA, sex, siblings, multiple birth, delivery method, maternal educational level, maternal asthma and atopy medication) and variables from the neonatal period (acute and chronic neonatal respiratory morbidity).

Observations with missing data in the explanatory variables in model 2 were censored in the analyses.

The model 2 analysis was also done on a subgroup from the later years with data available on maternal smoking during pregnancy to explore the effect of this potential confounder.

All data management, analyses and graphic illustrations were performed in SAS Statistical Software (version 9.4, SAS Institute, USA).

## Results

### Study population

**Fig 1:**
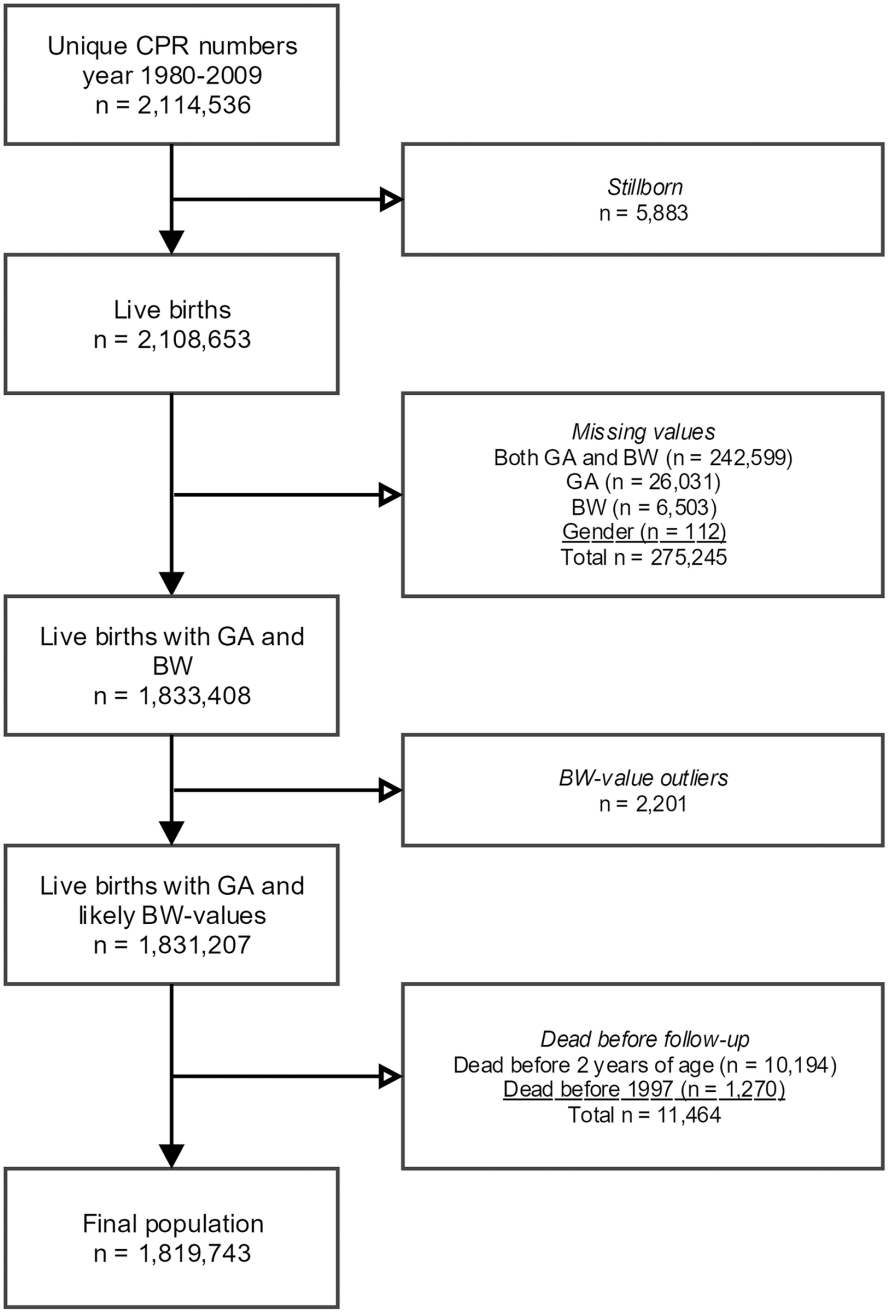
Flow-chart with exclusions from national birth cohort to final study population. CPR: Central Personal Registration, GA: gestational age, BW: birth weight.

A total of 1,819,743 individuals (86% of all registered births from 1980–2009) were included in the study population after the exclusion criteria described in Fig 1. The baseline characteristics are described in Table 1. BPD was linked to extreme prematurity (31.5% for GA 23–27 compared to 4.0% for GA 28–31, 0.04% for GA 32–36 and 0.0% for GA 37+), whereas acute neonatal respiratory disease also occurred in milder degrees of prematurity (18.2% in moderately preterm, 61.6% in very preterm and 77.0% in extremely preterm). Maternal factors such as asthma medication (5.1–6.2%), atopy medication (14.3–18.6%), and smoking (22.2–28.9%) showed smaller differences in distribution.

**Table 1:** Baseline characteristics of final study population in %.

		GA 37+ (n = 1,717,214)	GA 32–36 (n = 89,540)	GA 28–31 (n = 10,631)	GA 23–27 (n = 2,381)
Male sex		51.1	54.4	54.8	52.2
SGA		3.0	10.9	20.3	12.8
Cesarean section		13.9	39.4	65.6	54.0
Multiple birth		2.0	22.7	30.4	28.5
First born		44.8	53.7	58.2	60.5
BPD		0.0	0.04	4.0	31.5
Acute neonatal respiratory disease		1.7	18.2	61.6	77.0
Maternal educational level (ISCED)	0–2	37.6	40.2	40.1	41.3
	3–4	36.1	36.8	37.3	35.8
	5–8	26.3	23.0	22.7	22.8
Maternal asthma medication		5.1	5.4	6.2	5.6
Maternal atopy medication		18.6	15.9	14.7	14.3
Maternal smoking		22.2	26.9	28.9	26.7

The distribution of missing values in the study population is described in Table 2.

**Table 2:** Distribution of missing values in final study population in crude numbers (%).

		First born n (%)	Multiple birth n (%)	Cesarean section n (%)	Maternal educational level n (%)	Maternal smoking n (%)
Total		4906 (0.3)	21 (0.0)	2614 (0.1)	14,029 (0.8)	638,050 (35.1)
By birth year group	1980–1984	0 (0.0)	1 (0.0)	1845 (0.8)	3098 (1.3)	243,465 (100)
	1985–1989	1 (0.0)	4 (0.0)	433 (0.2)	3073 (1.1)	281,384 (100)
	1990–1994	0 (0.0)	2 (0.0)	127 (0.0)	2567 (0.8)	80,499 (24.8)
	1995–1999	562 (0.2)	13 (0.0)	91 (0.0)	1882 (0.6)	16,881 (5.1)
	2000–2004	609 (0.2)	1 (0.0)	109 (0.0)	1044 (0.3)	8466 (2.6)
	2005–2009	3734 (1.2)	0 (0.0)	9 (0.0)	2365 (0.7)	7355 (2.3)
By gestational age group	37+	4325 (0.3)	14 (0.0)	2419 (0.1)	13,253 (0.8)	605,081 (35.2)
	32–36	478 (0.5)	4 (0.0)	156 (0.1)	696 (0.8)	28,697 (32.1)
	28–31	87 (0.8)	0 (0.0)	33 (0.3)	68 (0.6)	3558 (33.5)
	23–27	16 (0.7)	3 (0.1)	6 (0.3)	12 (0.5)	714 (30.0)

### Statistical analyses

The extraction of data on the outcome purchase of prescription asthma medication yielded a total of 10,521,952 2-year period observations for the included 1,819,743 individuals, of which 299,728 (2.8%) 2-year periods were censored due to emigration or death. Of the non-censored observations, 448,728 (4.4%) were registered as positive for asthma medication.

**Fig 2:**
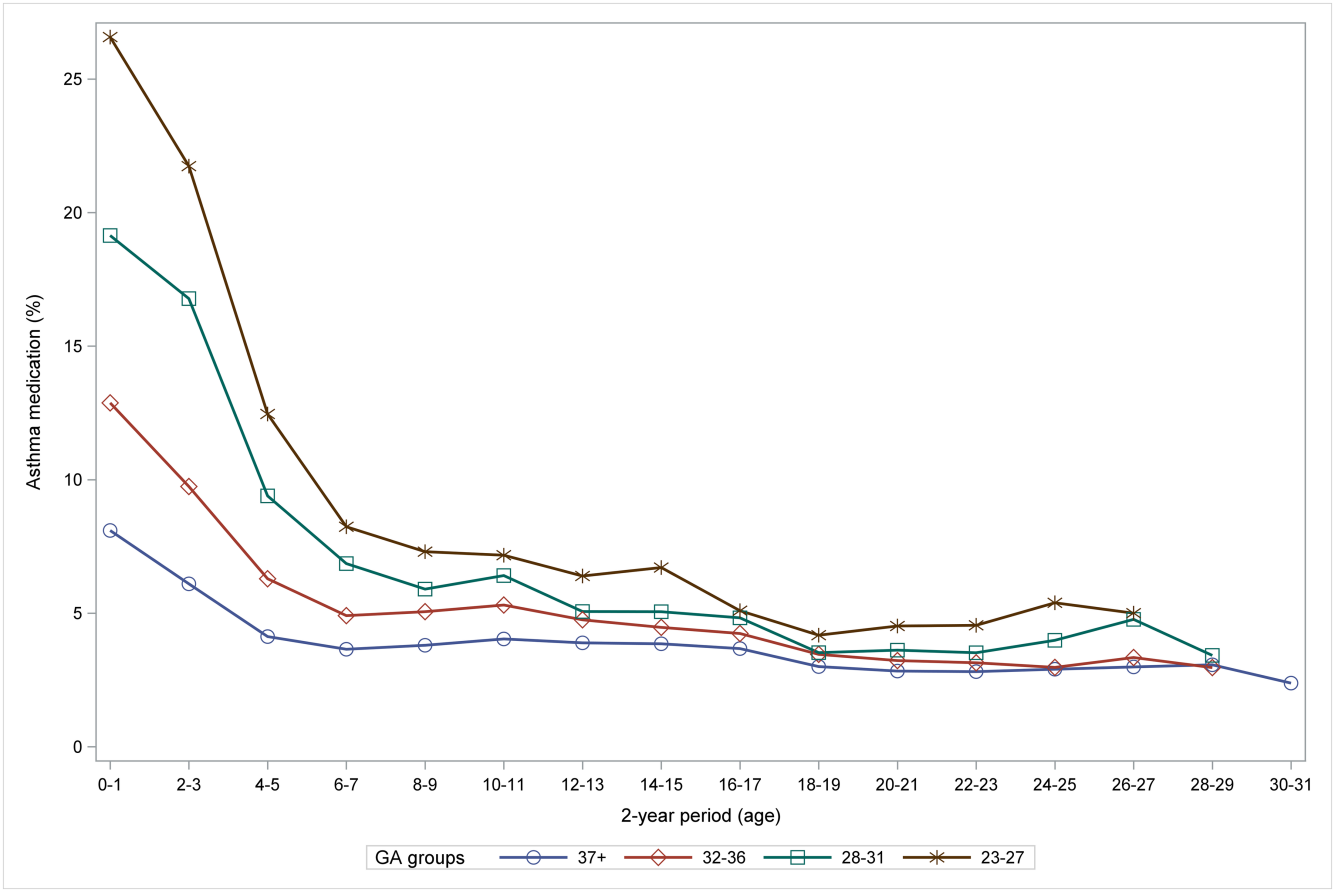
The inverse dose-response relationship between GA groups and asthma medication (in %) decreases and weakens with age. GA: gestational age.

The inverse dose-response relationship between GA and the crude rate of purchase of prescription asthma medication was consistent across all age groups, although the gradient decreased markedly during childhood (Fig 2).

**Fig 3:**
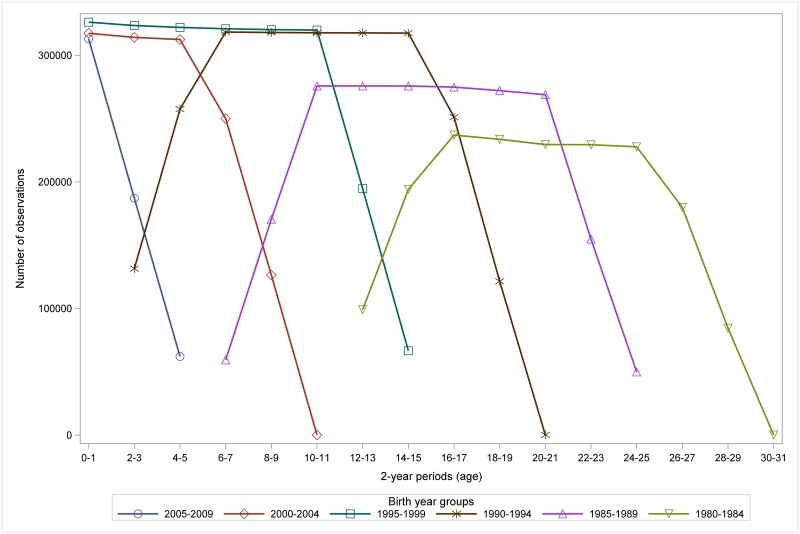
Distribution of number of observations on 2-year age periods by birth year groups. Illustration of the different follow-up age periods according to birth year during the follow-up period 1995–2011.

The number of observations varied among the birth cohorts and calendar years since medication data was only available from 1995 and considering the latest birth cohorts only had a limited period of observation (Fig 3).

**Fig 4:**
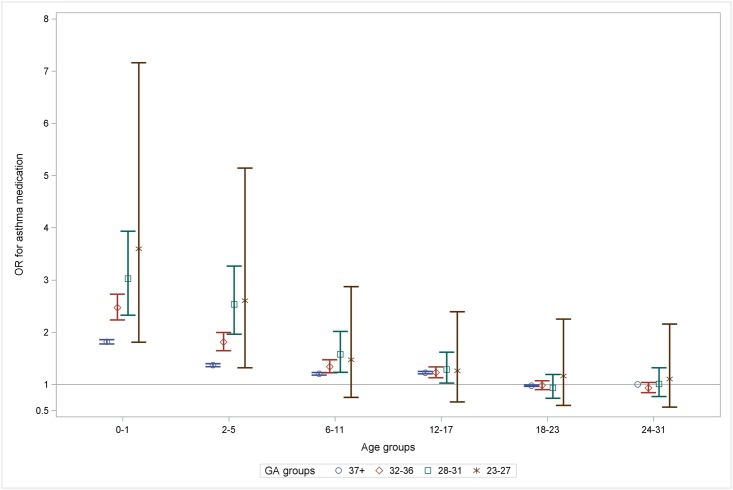
ORs for purchase of prescription asthma medication in age groups per GA-group illustrating gradual decrease of the GA-effect to loss of statistical significance in adulthood. The reference was GA 37+ at age 24–31. Adjusted for birth year, sex, SGA, maternal atopy and asthma medication, maternal educational level, cesarean section, older sibling, acute and chronic neonatal respiratory disease and interactions GA*age group and GA* birth year. OR: odds-ratio, GA: gestational age.

We first explored the effect of GA *per se* using the GEE model 2 with the GA groups as a categorical variable to allow for independent estimates of effects. The adjusted analysis supports the findings from our descriptive analyses, illustrating a higher odds-ratio (OR) for more preterm GA-groups until age 12–17 years, a gradual decrease in ORs with age and loss of statistical significance in early adulthood (Fig 4).

Next, GA was modelled as an ordinal variable, i.e. the GA groups as a variable with four steps. In Table 3, the main effect of age demonstrates that across the board, the risk of respiratory symptoms with asthma medication purchase decreases with age. Because the reference was set as 24–31 years for the age variable and years 1980–1984 for the birth year variable, the main effect of GA (p = 0.44 in model 2) corresponds to the effect of GA at 24-31-years in individuals born 1980–1984. In model 1, GA showed a marginally significantly increased risk of asthma medication purchase for the oldest generations (p-value = 0.04).

**Table 3:** ORs main analyses.

		Model 1		Model 2	
		OR (95% CI)	Type-III test (p-value)	OR (95% CI)	Type-III test (p-value)
GA (per increase in group)		1.09 (1.01–1.18)	0.04	0.97 (0.90–1.05)	0.44
Age in years (reference: 24–31 years)	0	1.33 (1.23–1.45)	<0.0001	1.34 (1.24–1.46)	<0.0001
	2–5	1.02 (0.94–1.10)		1.01 (0.93–1.09)	
	6–11	1.05 (0.98–1.14)		1.05 (0.97–1.13)	
	12–17	1.17 (1.09–1.26)		1.18 (1.09–1.26)	
	18–23	0.95 (0.89–1.02)		0.95 (0.89–1.01)	
Birth year (reference: 1980–1984)	1985–1989	0.96 (0.88–1.05)	<0.0001	0.96 (0.87–1.05)	<0.0001
	1990–1994	0.98 (0.89–1.06)		1.02 (0.93–1.11)	
	1995–1999	1.05 (0.96–1.14)		1.21 (1.11–1.31)	
	2000–2004	1.24 (1.14–1.35)		1.51 (1.38–1.64)	
	2005–2009	1.89 (1.73–2.06)		2.27 (2.08–2.48)	
Age * gestational age (reference: 24–31 years)	0	1.33 (1.23–1.43)	<0.0001	1.36 (1.26–1.46)	<0.0001
	2–5	1.33 (1.24–1.43)		1.36 (1.26–1.46)	
	6–11	1.14 (1.06–1.22)		1.15 (1.07–1.24)	
	12–17	1.04 (0.97–1.11)		1.04 (0.98–1.11)	
	18–23	1.03 (0.97–1.09)		1.04 (0.98–1.10)	
Birth year * gestational age (reference: 1980–1984)	1985–1989	1.02 (0.94–1.11)	0.02	1.02 (0.94–1.10)	0.001
	1990–1994	1.07 (0.99–1.16)		1.03 (0.95–1.11)	
	1995–1999	1.10 (1.02–1.19)		1.11 (1.03–1.20)	
	2000–2004	1.11 (1.03–1.20)		1.10 (1.02–1.19)	
	2005–2009	1.12 (1.04–1.21)		1.12 (1.04–1.21)	
Female sex		NA		0.72 (0.72–0.73)	<0.0001
Small for gestational age		NA		1.09 (1.06–1.12)	<0.0001
Maternal atopy medication		NA		1.37 (1.35–1.38)	<0.0001
Maternal asthma medication		NA		5.57 (5.48–5.65)	<0.0001
Maternal educational level (reference: ISCED 0–2)	ISCED 3–4	NA		1.00 (0.99–1.01)	<0.0001
	ISCED 5–8			0.94 (0.93–0.95)	
Cesarean section		NA		1.13 (1.12–1.15)	<0.0001
First born		NA		1.03 (1.02–1.04)	<0.0001
Acute neonatal respiratory disease		NA		1.24 (1.21–1.28)	<0.0001
BPD		NA		1.44 (1.28–1.63)	<0.0001

Estimates are adjusted for the other variables in the model.

OR: odds-ratio

95% CI: 95% confidence interval

NA: not applicable (not included in model)

*: interaction between separate links.

The interaction terms GA*age (p-value<0.0001) and GA*birth year (p-value 0.001) were significant overall. We found a substantial effect of GA in children below 12 years of age and from birth year 1995 (ORs 1.33, 1.33 and 1.14 for age groups 0, 2–5 and 6–11 respectively and 1.12, 1.11 and 1.10 for birth years 2005–2009, 2000–2004 and 1995–1999 respectively), while it was marginal for older individuals and earlier birth years (Table 3).

### Sub-group analyses

The sub-group-analysis on individuals with available data on maternal smoking during pregnancy demonstrated that this potential confounder had only little effect. The data consisted of 5,844,148 observations from 1,181,693 individuals born from 1991–2009 which were analyzed with the GEE model 2 both with and without the smoking variable (S2 Table). While maternal smoking showed an adjusted OR of 1.25 (95% confidence interval (CI) 1.24–1.27), inclusion of the variable did not alter the effect size of the other variables considerably. In agreement with main analyses, there was no significant effect of GA in the oldest group (18–19 years of age) and a steady decrease in risk of asthma medication purchase with age was observed. It is important to note, that this sub-group had almost complete outcome measurements from birth for all individuals, ie. they do not lack baseline measurements for the oldest generations.

## Discussion

In this unique longitudinal large dataset, we confirmed our previous findings from the cross-sectional evaluation of the association between GA and purchase of prescription asthma medication [5]: the strong association found between GA and purchase of prescription asthma medication in childhood weakens with age and largely disappears in adulthood. Importantly, our current findings imply that the weakening of this association did not seem to be due simply to a cohort effect: the interaction terms between GA and birth year were statistically significant only from 1995 and onwards. In the same manner, the interaction between GA and age was statistically significant up to 11 years of age only. The birth year variable did however show a doubling of asthma risk over the time period studied (OR 2.27 in 2005–2009, compared to 1980–1984), confirming the trend in asthma prevalences throughout the follow-up period [6].

The estimate for the crude risk in 24-31-year-olds was small: +9% per GA group (OR 1.09, 95% CI 1.01–1.18). The adjusted risk was independent of GA.

Although children born preterm have a documented higher risk of respiratory complaints [3], the decrease in association with GA during childhood might in part be related to improved clinical assessment by diagnostic tools such as lung function (spirometry), which are typically feasible from school age and onwards. Lung function assessment could indicate to the clinician that the respiratory complaints of individuals born preterm are not necessarily similar to classical atopic asthma. They would therefore often be unresponsive to such medication. Furthermore, some children born preterm may “outgrow” their respiratory symptoms, for example due to the catch-up alveolarization phenomenon [19]. In other words, the changes in asthma medication over time could either be due to increased availability of diagnostic tools and the ensuing more specific evaluation of treatment responses or to improved airway function with age–if not objectively then subjectively. It is interesting to note however, that new research points to a tracking of airway function in individuals born extremely preterm, revealing persistent substantial airflow limitation and impaired airway function, both in individuals with and without BPD [20,21].

The presence of a GA effect in young adults in model 1 (the crude risk), but absence in model 2 (adjusted for all available potential confounding and modifying variables) suggests that GA *per se* is not a significant risk factor for asthma medication purchase in adolescence and young adulthood. In fact, it seems that the increase in crude risk is explained by factors associated with the prenatal period or birth (maternal factors including atopic predispositions, smoking, mode of delivery, sex, birth weight), and by factors of the neonatal period (respiratory distress and BPD). Maternal use of atopy medication and asthma medication (OR 1.37 and 5.57 respectively) and neonatal respiratory disease (OR 1.24 for acute disease and 1.44 for BPD) seemed to be particularly strongly associated with the outcome. Put another way, these factors may play a more significant role in determining an individual’s risk of later pulmonary sequelae requiring anti-asthmatic treatment than the fact of being born preterm in itself. Individuals with these risk factors might still be at increased risk of impaired pulmonary function in both child- and adulthood.

The observational nature of the study does not permit an exploration of the causal pathways and BPD remains a major risk factor for persistent decreased lung function and pulmonary impairment, presumably throughout life, which emphasizes the need for a continuous follow-up in order to explore the risk for COPD later in life regardless of BPD type (“old” vs. “new”) [22–24].

The important strengths of the study are the inclusion of an almost complete nationwide birth cohort and the access to emigrational data and death both in the country and abroad, resulting in reliable censoring. The Danish National Prescription Registry provides high-quality data on all purchases of prescription medication: purchases are automatically registered from all out-of-hospital pharmacies and digitally reported to the Danish Health Authority, and the records form the basis for the Danish medication reimbursement system [25]. Data are directly linked with the patient’s CPR number. In-hospital pharmacies are not included in the database but have no sales. Therefore, only medications delivered at discharge would be missing in the database. Although the data were not originally collected for research purposes, all these factors provide excellent external validity with low risk of misclassification and loss of data. The concordance between questionnaire-based data and register data is high and has been studied specifically for asthma medication in children [26]. The only self-reported variable in the study was maternal smoking during pregnancy. The percentage of missing values was low and homogeneously distributed except for the smoking variable (available only from 1991 and onward). There are no records of personal smoking history available in the Danish registers.

The most important weaknesses are that the purchase of asthma and atopy medication was used as a proxy for asthma-like symptoms and atopic disease respectively. However, purchase of asthma medication has previously been validated as a good method for identifying asthmatic school children with a high specificity [27]. Furthermore, the definition of our outcome aimed at studying the more severe end of the disease spectrum. This strict outcome definition also deals with the unknown factor of patient compliance under the assumption, that a patient who has used a prescription more than once or used different drugs is considered more likely to have actively used the prescribed drug. Though the definition of fixed 2-year periods (starting every even birthday) may have lowered the prevalences further, we believe that it only contributed to increasing the outcome reliability. The beginning of medication registration in 1995 also resulted in a lack of baseline asthma medication data for the oldest generations of the study (Fig 3), which prevents a complete study of the trend in GA-effect in these individuals. However, agreement between our main analyses and sub-group analyses with baseline-measurements for almost all children implies that the lack of some baseline measurements in the main analysis should not affect the results. Similarly, it was not possible to include other potentially confounding factors such as maternal antibiotic use, which introduces the risk of residual confounding. Finally, as the chance of survival of extremely preterm infants was small [28], the oldest generations in our study had only few individuals born extremely preterm (0.1% of the study population) and constituted a smaller part of the study population due to growing birth numbers. The improvement of survival after preterm birth during the study period might negatively affect the generalizability of the study. This could be indicated by the interaction between GA and birth year, but since the youngest age groups of the study population have not yet reached adulthood, we cannot yet conclude on this aspect.

## Conclusion

In this longitudinal study, we did not find an association between GA and purchase of asthma medication in older age groups, consistent with our prior belief that the effect of GA towards the risk of asthma diminishes with age. It remains to be investigated if this association will recur in later adulthood and whether it is prematurity *per se* or rather neonatal respiratory disease that increases the risk of pulmonary impairment later in life.

## Supporting information

S1 Table RECORD Statement. *Reference: Benchimol EI, Smeeth L, Guttmann A, Harron K, Moher D, Petersen I, Sørensen HT, von Elm E, Langan SM, the RECORD Working Committee. The REporting of studies Conducted using Observational Routinely-collected health Data (RECORD) Statement. *PLoS Medicine* 2015; in press. * Checklist is protected under Creative Commons Attribution (CC BY) license.
(DOCX)

S2 Table ORs for sub-group analysis on maternal smoking using model 2 with and without inclusion of the variable. (DOCX)
