# Supplementary material for: The increased purchase of asthma medication for individuals born preterm seems to wane with age: A register-based longitudinal national cohort study
Source: PLoS One. 2018 Jul 5;13(7):e0199884. doi: 10.1371/journal.pone.0199884 (PMC6033435; doi:10.1371/journal.pone.0199884)
Supplement: S2 Table — (DOCX) [file pone.0199884.s002.docx]

**S2 Table. Subgroup analysis to explore the effect of the maternal smoking as a counfounder (OR and 95%-CI)**

|  | | Without maternal smoking included in analysis | With maternal smoking included in analysis |
| --- | --- | --- | --- |
| Maternal smoking | | - | 1.25 (1.24-1.27) |
| GA (per increase in group) | | 1.02 (0.88-1.17) | 1.01 (0.88-1.16) |
| Age in years (reference: 18-19 years) | 0 | 1.47 (1.26-1.71) | 1.47 (1.26-1.72) |
|  | 2-5 | 1.10 (0.94-1.28) | 1.10 (0.94-1.28) |
|  | 6-11 | 1.15 (0.98-1.34) | 1.15 (0.99-1.34) |
|  | 12-17 | 1.26 (1.09-1.46) | 1.26 (1.09-1.47) |
| Birth year (reference: 1991-1994) | 1995-1999 | 1.13 (1.07-1.20) | 1.15 (1.08-1.22) |
|  | 2000-2004 | 1.39 (1.31-1.48) | 1.42 (1.34-1.51) |
|  | 2005-2009 | 2.10 (1.97-2.23) | 2.16 (2.03-2.30) |
| Age * gestational age (reference: 18-19 years) | 0 | 1.33 (1.16-1.53) | 1.33 (1.16-1.53) |
|  | 2-5 | 1.34 (1.16-1.53) | 1.34 (1.17-1.53) |
|  | 6-11 | 1.12 (0.98-1.29) | 1.12 (0.98-1.29) |
|  | 12-17 | 1.02 (0.89-1.16) | 1.02 (0.89-1.16) |
| Birth year * gestational age (reference: 1991-1994) | 1995-1999 | 1.07 (1.01-1.13) | 1.07 (1.01-1.13) |
|  | 2000-2004 | 1.06 (1.01-1.12) | 1.07 (1.01-1.12) |
|  | 2005-2009 | 1.08 (1.02-1.14) | 1.08 (1.02-1.14) |
| Female sex | | 0.64 (0.63-0.64) | 0.64 (0.63-0.64) |
| Small for gestational age | | 1.12 (1.09-1.16) | 1.08 (1.04-1.11) |
| Maternal atopy medication | | 1.33 (1.31-1.35) | 1.34 (1.32-1.36) |
| Maternal asthma medication | | 3.77 (3.69-3.85) | 3.72 (3.64-3.80) |
| Maternal educational level (reference: ISCED 0-2) | ISCED 3-4 | 0.99 (0.97-1.00) | 1.01 (1.00-1.03) |
|  | ISCED 5-8 | 0.89 (0.88-0.90) | 0.94 (0.92-0.95) |
| Cesarean section | | 1.16 (1.14-1.17) | 1.16 (1.14-1.18) |
| First born | | 1.00 (0.99-1.01) | 1.00 (0.99-1.02) |
| Acute neonatal respiratory disease | | 1.26 (1.22-1.30) | 1.27 (1.23-1.31) |
| BPD | | 1.32 (1.16-1.50) | 1.33 (1.17-1.52) |
